# Cost‐Effectiveness of the I'm Ready HIV Self‐Testing Programme Among High‐Risk Populations in Canada

**DOI:** 10.1002/jia2.70097

**Published:** 2026-04-07

**Authors:** Lisa Masucci, Hawre Jalal, Sean B. Rourke, Kristin McBain, Min Xi, Wei Zhang, Hai V. Nguyen, William W. L. Wong, M. John Gill, Alice Zwerling, Kednapa Thavorn

**Affiliations:** ^1^ Ottawa Hospital Research Institute The Ottawa Hospital Ottawa Ontario Canada; ^2^ School of Epidemiology and Public Health University of Ottawa Ottawa Ontario Canada; ^3^ MAP Centre for Urban Health Solutions Unity Health Toronto (St. Michael's Hospital) Toronto Ontario Canada; ^4^ University of Toronto Toronto Ontario Canada; ^5^ Faculty of Pharmaceutical Sciences University of British Columbia Vancouver British Columbia Canada; ^6^ Memorial University St. John's, Newfoundland Canada; ^7^ School of Pharmacy University of Waterloo Kitchener Ontario Canada; ^8^ Cumming School of Medicine University of Calgary Calgary Alberta Canada

**Keywords:** acquired immunodeficiency syndrome, cost‐effectiveness, diagnostic tests, HIV acquisition, HIV self‐testing, point‐of‐care testing

## Abstract

**Introduction:**

While HIV self‐testing (HIVST) presents a promising solution for early HIV detection, access to such testing remains limited in Canada. Achieving the United Nations 95% target for HIV status awareness requires scalable and cost‐effective implementation approaches. The I'm Ready programme is a national, mail‐based HIVST initiative targeting key high‐risk populations supplemented by peer navigation supports to enhance engagement. This study aimed to explore the cost‐effectiveness of the I'm Ready programme from the perspective of Canada's publicly funded healthcare system.

**Methods:**

We developed a Markov model to predict the lifetime costs and quality‐adjusted life‐years (QALYs) for high‐risk individuals receiving HIVST through the I'm Ready programme compared to point‐of‐care testing in a physician's office (standard care). Probability and health utility values were obtained from published literature, while costs were obtained from the pilot I'm Ready programme or secondary Canadian data sources. Costs and outcomes were discounted 1.5% annually, with costs reported in 2024 Canadian dollars.

**Results:**

At a 53% uptake, 100% HIVST sensitivity and 99.5% specificity, the I'm Ready programme was associated with an incremental cost of C$270 and a QALY gain of 0.01 per person, with an incremental cost‐effectiveness ratio of $23,331/QALY compared to standard care. Key drivers of cost‐effectiveness included cost and utility associated with antiretroviral therapy initiation, utility of the AIDS health state and testing uptake under standard care.

**Conclusions:**

At the current test uptake and diagnostic accuracy levels, the I'm Ready programme is cost‐effective at the willingness‐to‐pay threshold of $50,000 per QALY. While findings reflect the Canadian health system context, this study offers broader insight into the value of HIVST as a public health tool to accelerate progress towards global HIV awareness targets.

## 1 Introduction

Sexually transmitted and bloodborne HIV acquisition continues to pose a significant public health concern globally [1]. Internationally, the UNAIDS 95–95–95 targets have been widely adopted to frame efforts to reduce HIV‐related morbidity and mortality, with the first target emphasizing the importance of HIV status awareness [2]. In Canada, progress towards reaching this goal has been challenged by persistent gaps in testing, monitoring and evaluation [3]. At the end of 2022, an estimate of 65,270 people were living with HIV, and 11% were unaware of their HIV status [4, 5]. These gaps may widen further as access to primary care physicians continues to decline nationwide [6].

Improving access to HIV testing remains a critical public health priority, particularly among populations disproportionately affected by HIV, including gay, bisexual and other men who have sex with men (gbMSM), people who inject drugs (PWID), Indigenous Peoples, and African, Caribbean and Black and other racialized communities. These populations experience substantially higher HIV incidence and prevalence than the general population and account for a disproportionate share of the overall disease burden [4].

People's knowledge of their HIV status, as well as their partner's, is crucial to increase diagnosis and reduce downstream HIV‐related morbidity and mortality [7]. The World Health Organization (WHO) highlights that the overarching goal of HIV testing services is to deliver a diagnosis and successfully facilitate access to and uptake of HIV prevention, treatment and care [7]. Standard lab‐based testing approaches with a primary healthcare provider may not be appropriate to reach many marginalized populations, as they may have trouble accessing testing, and they may feel discomfort disclosing their sexual history face‐to‐face with a provider [8]. Point‐of‐care testing (POCT) with the INSTI HIV‐1/HIV‐2 Antibody Test (INSTI, bioLytical Laboratories Inc.) has been available in Canada since 2005 [9]. This test has been available in primary care settings; however, uptake has been as low as 11% [9]. The WHO has called for the improvement of HIV testing and uptake by making testing available in locations and settings convenient to people from key populations and offering HIV self‐testing (HIVST) in order to increase access [7].

In November 2020, Canada approved the INSTI HIVST (Abbott Architect HIV Ag/Ab Combo test), which utilizes the same technology as POCT. In contrast to the POCT, the INSTI HIVST can be used without the assistance of a healthcare provider in a setting of the patient's choosing (e.g. at home). As such, this technology has the potential to address some common access barriers that are present in conventional testing approaches. An HIVST distribution project called the I'm Ready programme was implemented in Canada to distribute free HIVST kits across Canada. Although the cost‐effectiveness of HIVST has been explored in other countries [10, 11, 12, 13, 14, 15, 16], the cost‐effectiveness of this HIVST implementation strategy has not been established. This study was, therefore, conducted to identify the conditions under which the I'm Ready programme is cost‐effective.

## 2 Methods

### 2.1 I'm Ready Programme

The I'm Ready programme was a national implementation initiative project launched in Canada between June 2021 and 2023 to improve access to HIV testing among priority populations. The programme distributed free, mail‐based INSTI HIVST kits across the country and offered optional peer navigation to support linkage to care. Peer navigators provided secure, virtual assistance before, during or after testing, offering guidance on confirmatory testing, prevention and treatment [17]. Participants could also anonymously report their results through a mobile application, which provided tailored resources for next steps in care. The INSTI HIVST is a rapid, self‐administered test that delivers results under 60 s without the need for a healthcare provider [17, 18].

### 2.2 Model Structure and Study Population

We developed a probabilistic Markov model with an embedded decision tree and a monthly cycle length to explore the cost‐effectiveness of the I'm Ready programme compared to opportunistic POCT in a physician's office (standard care). The model structure was adapted from a previously published model [19] and consultations with clinical experts. Our analysis focused on adults aged 18 or older from populations at increased risk of HIV acquisition in Canada, including gbMSM, PWID, Indigenous Peoples, and African, Caribbean and Black communities. We simulated a cohort of individuals starting at age 18 and followed them over a lifetime horizon.

The model tracked individuals through six health states: HIV negative, HIV positive, HIV diagnosed on antiretroviral therapy (ART), HIV diagnosed not on ART, AIDS and death (Figure S1a,b). Individuals entered the model in either the HIV negative or HIV positive health states, with the initial distribution based on the HIV prevalence estimates for the priority populations [4].

We assumed annual HIV testing, with individuals who are HIV negative or undiagnosed receiving either self‐testing or POCT under standard care. Those who received a reactive (true positive or false positive) result could undergo confirmatory blood testing. Confirmed HIV‐positive individuals were eligible to initiate ART, and if treated, remained on ART without interruption. Individuals with a true negative result remained in the HIV negative health state. However, those with a false negative result eventually progressed to HIV or AIDS. HIV‐negative individuals were at risk of acquiring HIV each year, based on HIV incidence rates for relevant high‐risk groups. HIV acquisition risk was assumed to remain constant over time. Disease progression and mortality varied by diagnosis and treatment status.

### 2.3 Model Inputs

#### 2.3.1 Population‐Specific Prevalence and Incidence

The prevalence and incidence of HIV were based on estimates for gbMSM high‐risk populations, as this group represents the highest proportion of HIV cases in Canada, and for whom data are readily available. Estimates were drawn from a recent Public Health Agency of Canada report [4]. While gbMSM were used as the reference population for parameterization, we acknowledge these estimates may not generalize to other key populations prioritized by the I'm Ready programme.

#### 2.3.2 Effectiveness of the Implementation Strategies: Test Uptake and Linkage to Care

We defined the effectiveness of the I'm Ready programme as the relative difference in the HIV test uptake compared to standard care. The baseline uptake of POCT in a physician's office was estimated from a discrete choice experiment involving 5113 members of the general public [20]. Results were stratified by risk group, and uptake estimates for high‐risk populations were used in the analysis. The study examined the overall predicted uptake of testing among five risk groups: under 25 years, gbMSM, African, Caribbean and Black, PWID, and risky sexual activities [20]. The effectiveness of the I'm Ready programme on HIVST uptake was based on a network meta‐analysis comparing web‐based ordering and mail‐based HIVST delivery strategies to facility‐based strategies [21] (Table 1). The analysis included eight studies, predominantly among gbMSM and transgender populations, and assessed three HIVST distribution strategies [21].

**TABLE 1:** Model input parameters.

Parameter	Mean value (standard error)	Distribution for PSA	Source
*Probabilities*			
HIV prevalence for high‐risk populations	0.07 (0.009)	Beta	[4]
HIV incidence for high‐risk populations	0.00014 (0.00002)	Beta	[4]
POCT in a physician's office			
Sensitivity of POCT (HIV 1/2 Antibody)	0.998 (0.0028)	Beta	[26]
Specificity of POCT (HIV 1/2 Antibody)	0.998 (0.0032)	Beta	[26]
Proportion of patients tested with POCT	0.34 (0.04)	Beta	[20]
HIVST			
Sensitivity of HIVST	1.00 (0.14)	Fixed	[18]
Specificity of HIVST	0.995 (0.003)	Beta	[18]
Risk ratio of a mail‐based HIVST distribution on HIV testing uptake versus standard care	1.55 (1.01)	Log‐normal	[21]
Confirmatory BT			
Proportion receiving confirmatory blood testing	0.85 (0.08)	Beta	[22, 23]
Relative risk of receiving confirmatory testing for HIVST versus standard care	0.83 (0.05)	Log‐normal	[24]
Sensitivity of confirmatory BT (Geenius HIV 1/2 confirmatory test)	1.00 (0.0015)	Fixed	[28]
Specificity of confirmatory BT (Geenius HIV 1/2 confirmatory test)	1.00 (0.0038)	Fixed	[28]
ART			
Proportion placed on ART	0.87 (0.02)	Beta	[4]
Progression			
Progression from HIV to AIDS without ART	0.022 (0.003)	Beta	[29, 30, 31, 32, 33, 34, 35]
Progression from HIV to AIDS with ART	0.006 (0.001)	Beta	[29, 31, 32, 33, 34, 35]
Mortality			
Standardized mortality ratio with ART	2.08 (0.68)	Log‐normal	[37]
Mortality hazard ratio without ART	7.02 (1.59)	Log‐normal	[38]
Frequency of HIV testing	Annual	Fixed	
*Cost, monthly $ per patient (2024 Canadian dollars)*			
I'm Ready programme cost (upfront, one‐time cost)	21.27 (2.71)	Gamma	[39]
I'm Ready monthly programme costs	1.26 (0.13)	Gamma	[39]
HIVST kit	43 (5.53)	Gamma	[18, 39]
POCT plus physician visit	92 (11.71)	Gamma	[29]
Confirmatory BT plus physician visit	135 (17.24)	Gamma	[29, 40]
HIV care off ART	371 (47.38)	Gamma	[41]
HIV care on ART	1750 (223)	Gamma	[41]
AIDS care	2558 (326)	Gamma	[41]
Cost of transitioning to death	5685 (1278)	Gamma	[43]
*Utilities, monthly*			
HIV negative	0.073	Fixed	[45]
HIV (undiagnosed)	0.066 (0.008)	Beta	[46]
HIV diagnosed on ART	0.069 (0.009)	Beta	[46]
HIV diagnosed off ART	0.060 (0.008)	Beta	[46]
AIDS	0.058 (0.007)	Beta	[47]
Dead	0		

Abbreviations: ART, antiretroviral therapy; BT, blood test; HIVST, HIV self‐test; POCT, point‐of‐care test; PSA, probabilistic sensitivity analysis.

We defined linkage to care as the proportion of individuals with a reactive test result who subsequently underwent confirmatory blood testing with a healthcare provider. Confirmatory testing is required to initiate ART and is conducted via venous blood draw. Based on several studies reporting 70%–100% of linkage rates [22, 23], we used an average of 85% from these studies as the base case estimate [22, 23]. We assumed that the probability of linking to care (i.e. confirmatory blood testing) was different among the strategies [24]. This assumption was supported by another meta‐analysis suggesting that linkage to care is lower following HIVST compared to facility‐based testing, with a pooled risk ratio of 0.83 (95% CI 0.74, 0.92) [24]. The studies included in the analysis were among gbMSM, transgender and female sex worker populations [24]. We assumed ART initiation rates after confirmatory diagnosis were equivalent for both testing strategies, consistent with the requirement for healthcare involvement prior to treatment initiation. These proportions were obtained from a 2020 Public Health Agency of Canada report [4].

#### 2.3.3 Testing Accuracy

Diagnostic performance of the INSTI HIVST was obtained from a Canadian study comparing self‐testing outcomes to laboratory‐based reference standards [25]. The INSTI POC HIV‐1/HIV‐2 Rapid Antibody Test is administered by a healthcare professional and delivers results within 60 s using fingerstick or venous whole blood [26]. The accuracy of the POCT test was obtained from a study using the test on individuals with confirmed HIV‐1 and those without HIV [27]. The sensitivity and specificity of the POCT was 0.998 and 0.998, respectively. Confirmatory testing accuracy was based on the Geenius HIV 1/2 Confirmatory Assay (Bio‐Rad Laboratories), as reported by the manufacturer [28].

#### 2.3.4 HIV Progression and Mortality

Progression from undiagnosed HIV to AIDS in individuals not on ART and ART was informed by two published cost‐effectiveness studies [29, 30], one of which [30] derived disease progression based on published clinical studies [31, 32, 33, 34, 35]. All‐cause mortality among HIV‐negative individuals was derived from 2020−2022 Canadian life tables [36]. For those living with HIV, we applied elevated mortality risks based on treatment status: 2.08 times for those on ART [37] and 7.02 times higher for those not on ART [38].

#### 2.3.5 Costs of the I'm Ready Programme

Programme costs included both fixed upfront and recurring monthly costs. Upfront costs consisted of equipment and software (e.g. computers, website and app development), promotional campaigns (Facebook and Google Ads) and personnel (e.g. project management and training). Recurring costs included peer navigator support, follow‐up, test logistics and coordination. These costs were averaged across the 18,436 individuals who enrolled in the I'm Ready programme to receive the HIVST kit [39]. The per‐person upfront cost was $21.27, and the monthly recurring cost was $1.26 for individuals who were HIV negative or undiagnosed. The cost of the HIVST kit included the manufacturer's price and mailing costs [18].

#### 2.3.6 Costs of Standard Care and Confirmatory Testing

For individuals in standard care, total testing costs included the POCT kit and physician visit [29, 40]. In both strategies, those with reactive test results were eligible for confirmatory testing, including the Geenius HIV assay and an associated HIV medical visit [29, 40].

#### 2.3.7 Costs of HIV and AIDS

Health state costs (Table 1) were obtained from a published retrospective costing study at the Southern Alberta Clinic in Calgary, Alberta, Canada, which reported HIV‐related care costs over time [41]. Original costs (2017 Canadian dollars) were inflated to 2024 values using the consumer price index for healthcare services [42]. We also included the cost associated with transitioning to death from any health state [43]. All future costs were discounted at 1.5% annually (0.12% per month) as recommended by current Canadian economic evaluation guidelines [44].

#### 2.3.8 Health State Utilities and Quality‐Adjusted Life‐Years

Each health state was assigned a utility weight to estimate quality‐adjusted life‐years (QALYs). A utility of 1.0 represents perfect health, while a 0.0 indicates death. For those in the HIV negative health state, we assigned the general Canadian population utility weight [45]. The monthly utility values were specific to the HIV and AIDS health states (Table 1) [46, 47]. All QALYs were discounted 1.5% annually (0.12% per month) as recommended by current Canadian economic evaluation guidelines [44].

#### 2.3.9 Probabilistic Sensitivity Analysis

For the base case, we conducted a probabilistic analysis to characterize the uncertainty in the model inputs. Parameters were sampled from appropriate distributions; beta distributions for probabilities and utilities, log‐normal for relative risks and gamma for costs. Where available, 95% confidence intervals informed distributional parameters; otherwise, we applied +/− 25% from their base case value to define plausible ranges. We sampled the inputs randomly 10,000 times from the assigned distributions to estimate the mean outcomes. The likelihood of each strategy being more favourable across a range of willingness‐to‐pay (WTP) thresholds was presented as cost‐effectiveness acceptability curves (CEACs). We used a cost‐effectiveness threshold of C$50,000 per QALY, as it is commonly used by Canada's Drug Agency [44]. All analyses were conducted using R version 2024.04.2 and the Twig package [48, 49].

#### 2.3.10 Deterministic Sensitivity Analysis

We conducted a series of one‐way deterministic sensitivity analyses on all model inputs to evaluate the influence of individual model inputs on the incremental cost‐effectiveness ratios (ICERs). We varied each input parameter independently according to its 95% confidence intervals or +/− 25% from its base case value. Results were summarized using a tornado diagram based on incremental net benefit (INB), calculated using a WTP of C$50,000 per QALY. The I'm Ready programme was deemed cost‐effective if INB > 0 and not cost‐effective if INB < 0.

#### 2.3.11 Scenario Analysis

Scenario analyses were conducted to explore structural and contextual uncertainties. We performed multiple scenario analyses varying key assumptions, including: the relative impact of a mail‐based HIVST distribution on HIVST testing uptake versus standard care, cost of the I'm Ready programme, cost of the HIVST kit, testing frequency, starting age of the cohort and time horizon. In addition, we conducted threshold analyses to identify values at which the programme ceased to be cost‐effective. These thresholds were assessed for HIVST uptake, I'm Ready programme cost, HIVST kit cost and POCT uptake using the C$50,000 per QALY benchmark.

#### 2.3.12 Validation and Reporting

We assessed model validity using the economic model validation tool developed by Canada's Drug Agency [50]. We reviewed the results of the one‐way sensitivity analysis to ensure that the results were consistent with what we would expect when changing the values. The face validity of the model structure, assumptions, data and results were validated with clinical experts.

Reporting adhered to the Consolidated Health Economic Evaluation Reporting Standards (CHEERS) guideline for cost‐effectiveness evaluations [51]. To enhance relevance and inclusivity, study findings were reviewed by individuals with lived experience of HIV. Their feedback contributed to the interpretation of results and ensured alignment with the perspectives and priorities of affected communities.

#### 2.3.13 Ethics

This modelling exercise was based on previously published literature or reports and did not involve any human subject research. Therefore, research ethics board approval and consent were not required for this study.

## 3 Results

### 3.1 Base Case Analysis

**FIGURE 1:**
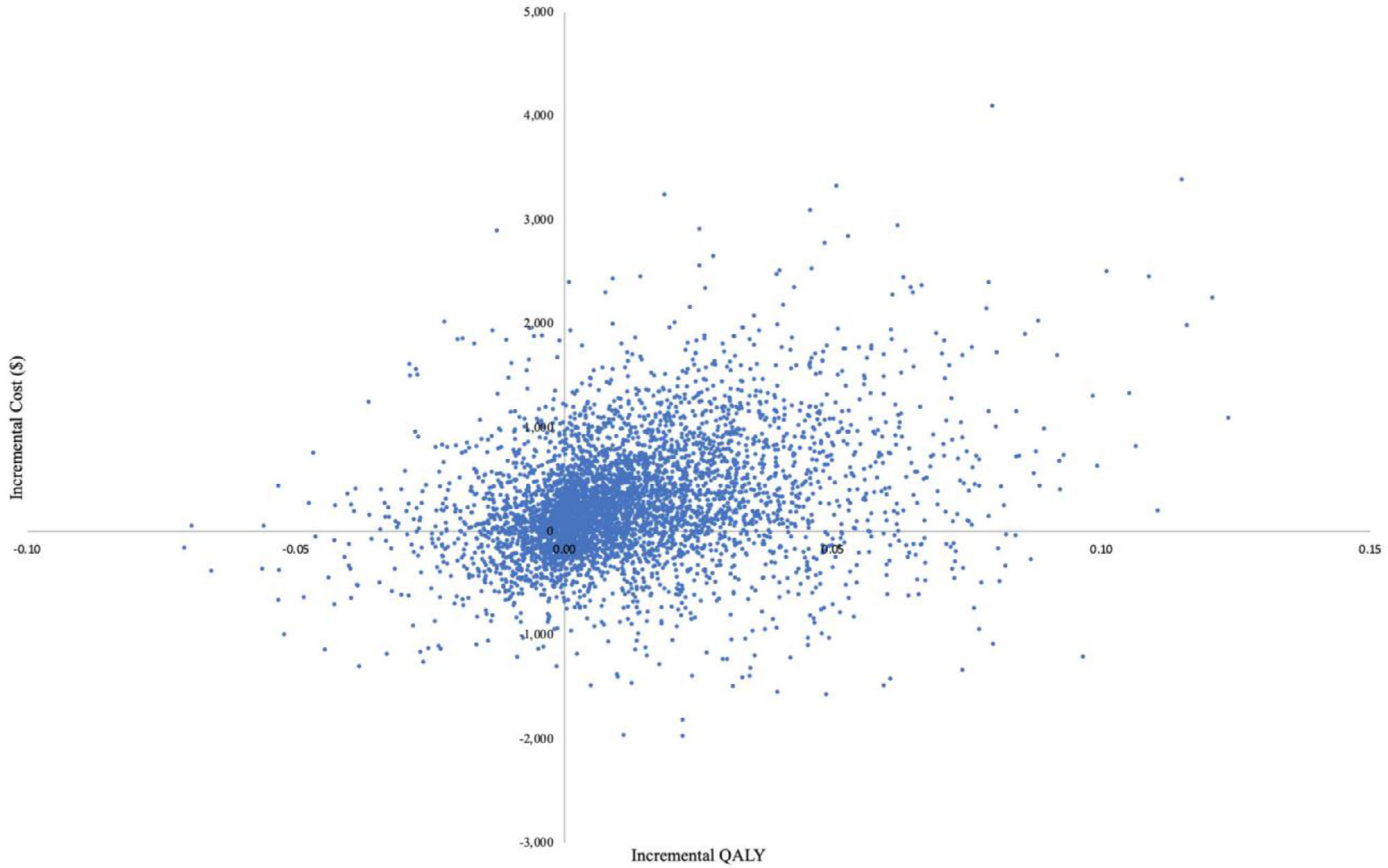
Cost‐effectiveness plane of the I'm Ready programme compared to POCT in a physician's office.

**FIGURE 2:**
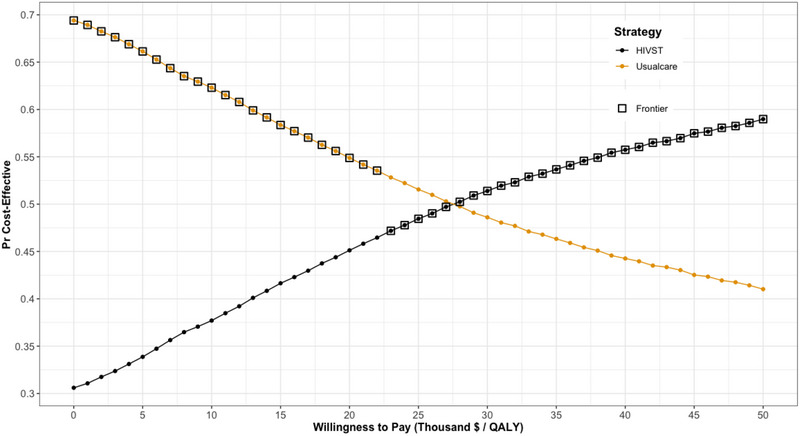
Cost‐effectiveness acceptability curves.

Over a lifetime horizon, the I'm Ready programme was associated with a higher cost (C$114,435 vs. C$114,165) and slightly improved QALYs (34.565 vs. 34.554) compared to standard care (Table 2). This led to an incremental ICER of C$23,331 (95% CI $23,011, $23,650). At a WTP threshold of $50,000/QALY, the incremental net benefit was C$309 (95% CI $289, $329), indicating that the programme is cost‐effective under a commonly accepted threshold. The probabilistic analysis also demonstrated that 76% of the 10,000 simulations fell in the northeast quadrant of the cost‐effectiveness plane, indicating that the I'm Ready programme was more costly and more effective than standard care (Figure 1). The CEAC demonstrated that the I'm Ready programme had a 59% chance of being cost‐effective at a WTP threshold of C$50,000/QALY (Figure 2).

**TABLE 2:** Base case results costs and health outcomes per patient.

	Total cost ($, disc)	Incr. cost ($, disc)	Total cost ($, undisc)	Incremental cost ($, undisc)	QALY (disc)	Incr. QALY (disc)	QALY (undisc)	Incr. QALY (undisc)	Life‐years (disc)	Incr. life‐years (disc)	Life‐years (undisc)		Incr. life‐years (undisc)
Standard care	114,165	270	189,179	415	34.554	0.011	53.982	0.017	40.153	0.005	62.793	0.009	
I'm Ready programme	114,435		189,594		34.565		53.999		40.158		62.802		
ICER (incremental cost per QALY, discounted)								C$23,331 (95% CI $23,011, $23,650)					
Incremental net benefit at C$50,000/QALY										C$309 (95% CI $289, $329)			

Abbreviations: Disc, discounted; incr, incremental; QALY, quality‐adjusted life‐years; undisc, undiscounted.

We also examined intermediate outcomes per 1000 individuals. The I'm Ready programme resulted in more HIV tests (21,321 vs. 13,735) and confirmatory tests (119 vs. 72) compared to standard care. In addition, the programme slightly reduced AIDS progression, with 8.79 cases per 1000 individuals in the programme and 8.86 for the standard of care.

### 3.2 Deterministic Sensitivity Analysis

**FIGURE 3:**
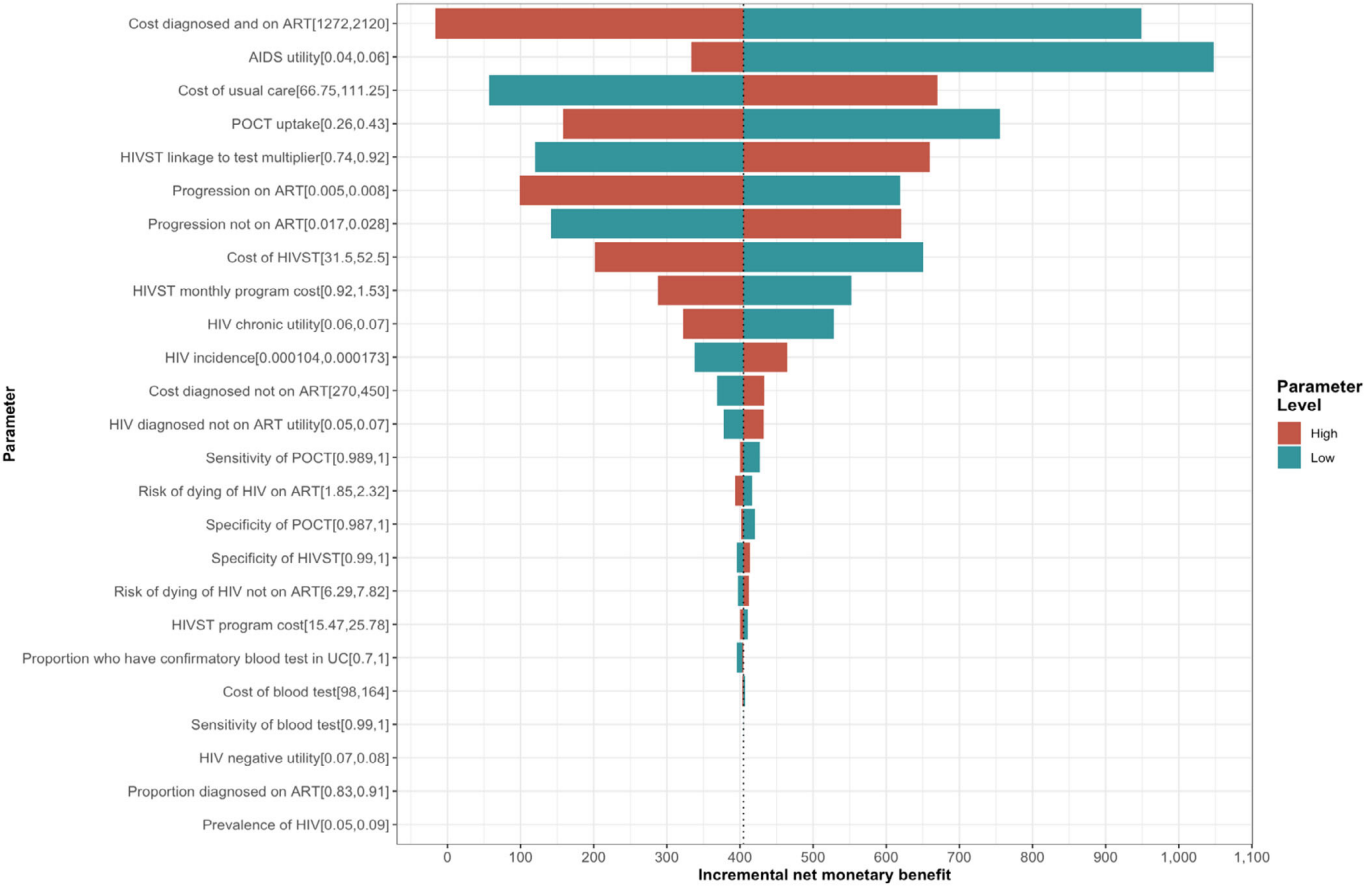
One‐way deterministic sensitivity analysis results.

Figure 3 demonstrates the variables that impacted the results. The five variables with the greatest influence on the model were: cost of initiating ART, the utility of being diagnosed and on ART, uptake of standard care testing, the cost of standard care and linkage to care with the HIVST. When the cost of initiating ART increased to $2120, the INB decreased to −$16. A decrease in the utility of the AIDS health state of 0.04 led to an INB of $1048. Reducing the linkage to care with the I'm Ready programme from 0.83 to 0.74 resulted in a $120 decrease in INB. When standard care testing uptake was reduced to 0.26, the INB increased to $307. Lastly, if the cost of standard care was reduced to $67, the INB would be reduced to $60. The results remained robust across all other one‐way sensitivity analyses, suggesting the model's conclusions are generally stable under parameter uncertainty.

### 3.3 Threshold Analysis

**FIGURE 4:**
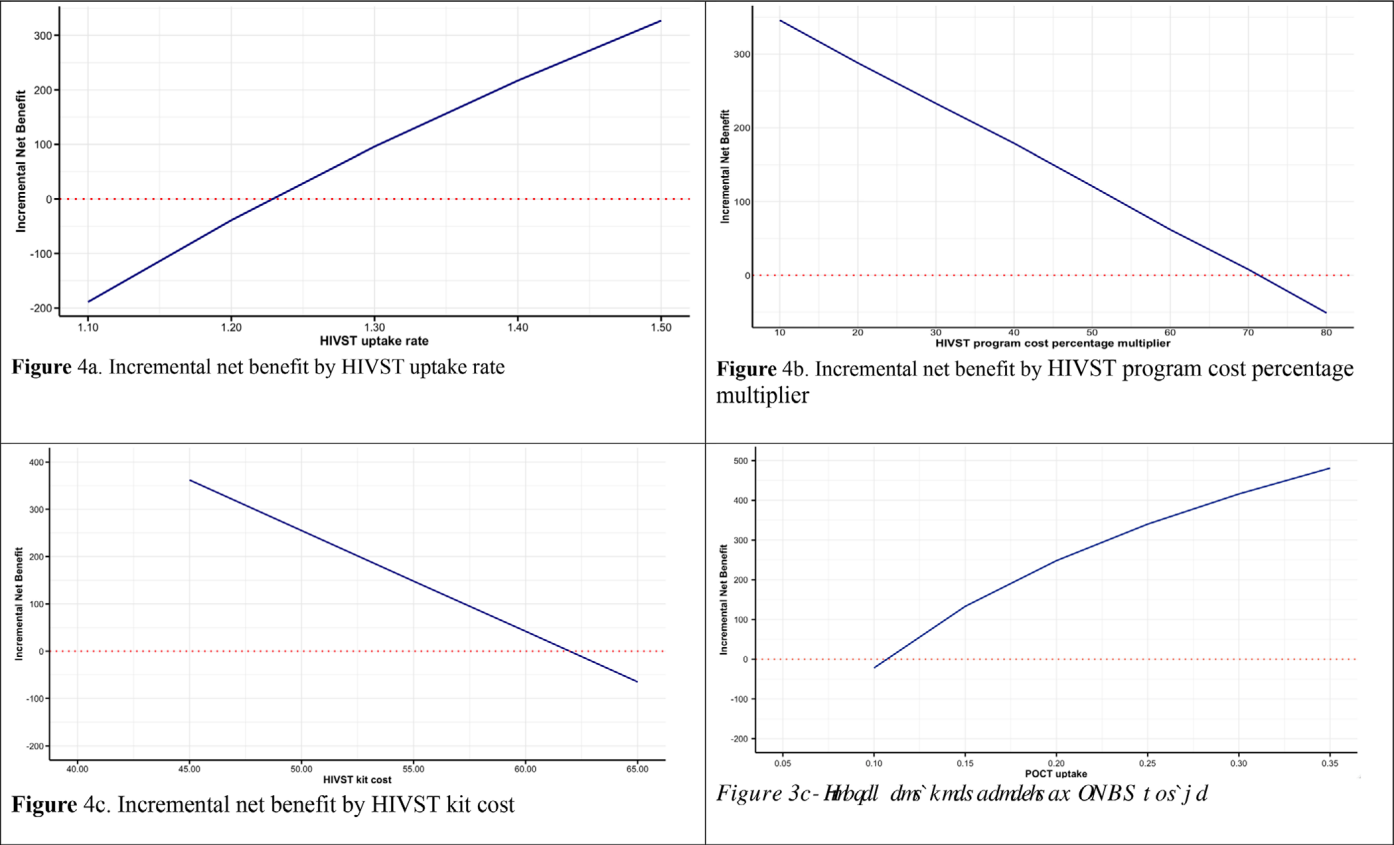
Threshold analysis.

We found that at an HIVST relative uptake rate of 1.21 or lower, the programme was no longer considered cost‐effective (INB = −$7) (Figure 4a). If the I'm Ready programme cost increased by 72% of the total programme cost, the I'm Ready programme was no longer considered cost‐effective (INB = −$51) (Figure 4b). If the HIVST kit cost increased to $62 per kit, the I'm Ready programme was no longer cost‐effective (Figure 4c). Finally, if the POCT uptake was reduced to 10.4%, the I'm Ready programme was no longer cost‐effective (Figure 4d).

### 3.4 Scenario Analysis

Scenario analysis results are presented in Table S1. Among all scenarios examined, the cost‐effectiveness of the I'm Ready programme was most sensitive to the analytic time horizon and the relative HIVST uptake rate. Reducing the time horizon from lifetime to 10 years led to an INB of −$11. The I'm Ready programme would not be considered cost‐effective using a WTP threshold of $50,000/QALY gained.

## 4 Discussion

This cost‐effectiveness analysis examined the conditions under which the I'm Ready programme would be cost‐effective. Under base‐case assumptions, including an HIVST uptake of 53% consistent with similar implementation strategies, the I'm Ready HIVST programme was cost‐effective compared to POCT delivered in a physician's office from the perspective of a publicly funded healthcare system. The programme yielded modest QALY gains at a relatively low incremental cost, with an ICER well below the commonly cited WTP threshold of C$50,000 per QALY. The primary mechanism driving these results was increased case identification through higher testing uptake. Once individuals were diagnosed and initiated on ART, treatment effectiveness and persistence were assumed to be equivalent across strategies due to the absence of empirical evidence that ART outcomes differ by testing modality. Accordingly, results were most sensitive to parameters related to testing uptake, ART costs and utilities, and health‐state utilities for advanced HIV and AIDS, highlighting the importance of diagnosis and linkage to care in determining the value of HIVST programmes.

Threshold analyses further underscore the conditional value of the I'm Ready programme. The programme was no longer cost‐effective when the relative HIVST uptake rate declined to 1.21, when total programme costs increased by more than 72%, when the HIVST kit cost exceeded C$62 or when the POCT uptake was reduced to 10.4%. These findings identify boundaries within which the programme remains economically attractive and provide decision‐relevant information for procurement and implementation planning.

We also explored structural uncertainty related to time horizon and scale. Reducing the analytic time horizon from a lifetime to 10 years resulted in a negative incremental net benefit, reflecting the fact that the health and cost benefits of earlier HIV diagnosis accrue primarily in the long term through avoided AIDS cases and downstream cost offsets. Conversely, economies of scale may improve cost‐effectiveness as fixed upfront programme costs are spread across a larger number of users. A scenario analysis reducing the per‐person upfront cost from $21.27 to $15 had minimal impact on the ICER, suggesting that while scale effects could modestly improve economic attractiveness, the overall conclusions are robust to plausible variation in programme costs.

Our findings align with the broader economic literature on HIVST. Seven previous economic studies have assessed the value of HIVST across diverse settings and populations [10, 11, 13, 14, 15, 16, 52]. Five studies were conducted in Africa and found HIVST to be cost‐effective [10, 11, 13, 16, 52]. Two studies [14, 15] evaluated the eSTAMP, a US‐based mail‐out HIVST initiative paired with counselling, prioritizing gbMSM. Islam et al. found eSTAMP to be cost‐effective compared to the standard of care (i.e. no HIVST kits distributed), with the INBs ranging from −US$145 to US$631 USD [15] and a cost‐effective threshold of US$9365 per one additional diagnosis [15]. A subsequent trial‐based evaluation by the same team found that eSTAMP yielded more QALYs (14.86) and lower costs (cost‐savings of $74,476 USD) compared to standard care [14]. While differences in health system context, modelling approaches and inclusion of transmission dynamics limit direct comparability, our study reinforces the conclusion that HIVST programmes prioritizing key populations can provide good value for money in high‐income settings.

### 4.1 Public Health Policy Implications

HIVST has the potential to improve access to testing by reaching individuals who face structural, social or logistical barriers to facility‐based services. An earlier diagnosis enables timely ART initiation, reduces immunologic decline and lowers the risk of onward transmission. While UNAIDS 95‐95‐95 targets provide important context for HIV testing strategies, this analysis was not designed to project progress towards these targets. Rather, it evaluates the economic value of HIVST through its impact on diagnosis, health outcomes and costs—metrics directly relevant to resource allocation decisions. Given the current levels of test uptake and diagnostic accuracy, the I'm Ready programme may represent a viable option to augment existing HIV testing strategies in Canada and other jurisdictions with similar healthcare systems.

### 4.2 Limitations

Several limitations warrant consideration. First, baseline testing uptake varies across jurisdictions and care settings [53]. Standard‐of‐care uptake was informed by a discrete choice experiment conducted in the Canadian general population, while HIVST uptake estimates were drawn from studies among gbMSM. To address this concern, we conducted threshold analyses varying standard‐of‐care uptake and found that if the clinic‐based POC testing were to be reduced substantially, the HIVST would no longer be cost‐effective. Second, due to data limitations, we were unable to explicitly model heterogeneity across key populations. While I'm Ready prioritizes multiple groups, incidence and prevalence estimates primarily reflect data from gbMSM, which may limit the generalizability of study results. Third, we did not model ART interruptions, adherence or long‐term retention, which may influence the downstream effectiveness of testing strategies. While HIVST may influence linkage to care, there is insufficient evidence to support differential long‐term ART outcomes by testing modality. Fourth, HIV acquisition risk was assumed to remain constant over time. If HIV incidence declines, the cost per QALY gained would be expected to increase, making testing programmes appear less cost‐effective. Conversely, the model does not incorporate HIV transmission dynamics, which would likely strengthen the economic case for HIVST by capturing secondary prevention benefits. Fifth, costs were estimated from a publicly funded healthcare system perspective; patient‐incurred costs such as travel and productivity losses were excluded. Inclusion of these costs under a societal perspective would likely further favour HIVST. Finally, as an early economic evaluation, this study relied on indirect evidence to parameterize HIVST uptake rather than real‐world data from the I'm Ready programme. The comparative effectiveness of the I'm Ready programme on HIV testing uptake and linkage to care remains uncertain. Future studies should assess its real‐world impact; however, our analysis provides a transparent and policy‐relevant assessment of its cost‐effectiveness using the best available evidence.

### 4.3 Strengths

This is the first study to assess the cost‐effectiveness of a mail‐based HIVST implementation strategy in Canada. In the absence of trial‐based data, we employed a comprehensive modelling approach, incorporating extensive one‐way, probabilistic and scenario analyses to characterize uncertainty and identify conditions under which the programme provides value. This focus on threshold conditions is particularly valuable for policy‐makers and programme managers tasked with budgeting, procurement and scale‐up decisions. Additionally, our use of established economic modelling and validation tools further enhances the credibility and transparency of our findings.

## 5 Conclusions

The I'm Ready programme is a cost‐effective strategy to expand access to HIV testing in Canada. As countries work towards the United Nations 95‐95‐95 targets, expanding access to HIVST offers a practical, scalable and economically viable option to improve diagnosis rates and linkage to care, particularly in high‐risk populations. Our findings support the integration of HIVST into national testing strategies and provide a framework for future evaluations of real‐world HIVST implementation.

## Author Contributions

LM, KT, AZ and SBR formulated the research questions and conceptualized the study. KT, AZ, SBR, KM, HVN and WW secured research funding. LM and HJ programmed and validated the decision‐analytic model, and LM conducted the modelling analyses. LM and KT drafted the initial manuscript, and KT, AZ, HJ, SBR, KM, HVN, WWLW, MX and MJG critically revised it for important intellectual content. All authors reviewed and approved the final version for publication.

## Conflicts of Interest

The authors declare that they have no conflicts of interest.

## Supporting information




**Table S1**: Scenario analysis (deterministic), per patient
Figure S1a: Markov model
Figure S1b: Markov model with embedded decision tree

## Data Availability

The data that support the findings of this study are available from the corresponding author upon reasonable request.
