# Supplementary material for: Cost‐Effectiveness of the I'm Ready HIV Self‐Testing Programme Among High‐Risk Populations in Canada
Source: J Int AIDS Soc. 2026 Apr 7;29(4):e70097. doi: 10.1002/jia2.70097 (PMC13054515; doi:10.1002/jia2.70097)
Supplement: Supplementary file 1 — Table S1: Scenario analysis (deterministic), per patient Figure S1a: Markov model Figure S1b: Markov model with embedded decision tree [file JIA2-29-e70097-s001.docx]

**Table 1** Scenario analysis (deterministic), per patient

| **No.** | **Scenario** | **Additional cost (discounted)** | **QALY gained (discounted)** | **Incremental net benefit** |
| --- | --- | --- | --- | --- |
|  | **Base case** | **$270** | **0.01158** | **$309** |
| 1 | Lower cost of the HIVT kit $16 vs. $43 | (320) | 0.01 | Intervention dominates usual care |
| 2 | Testing frequency every 3 m vs. annual | ($429) | 0.01 | Intervention dominates usual care |
| 3 | Start age of cohort from 18 years to 25 years | $283 | 0.013 | $388 |
| 4 | Reducing the time horizon from lifetime to 10 years | $201 | 0.00379 | -$11 |

**
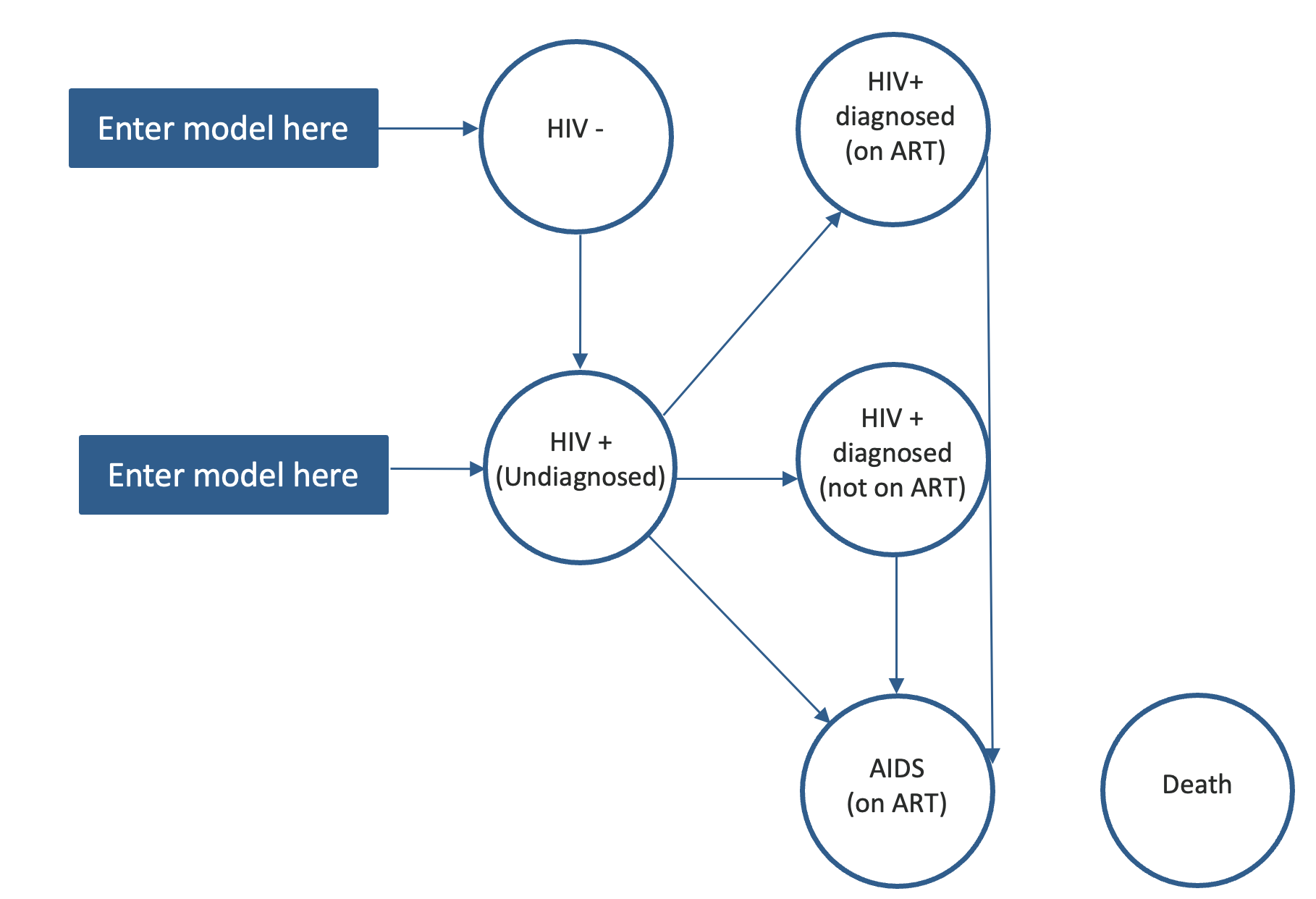
**

**Figure 1a**. Markov model


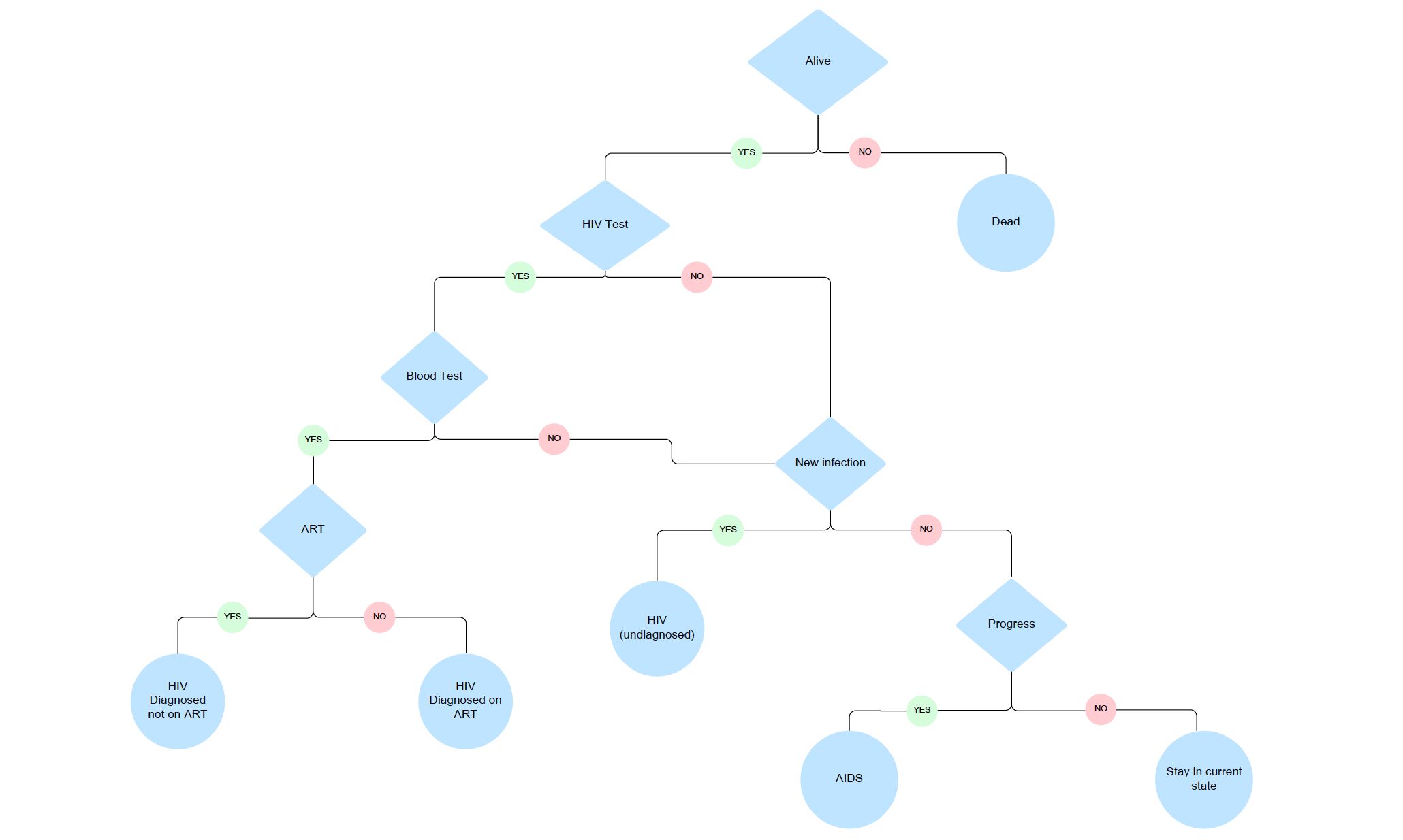


**Figure 1b.** Markov Model with embedded decision tree
